# BDSM and masochistic sexual fantasies in women with borderline personality disorder: simply on the spectrum of “normality” or source of suffering?

**DOI:** 10.1186/s40479-025-00283-6

**Published:** 2025-02-22

**Authors:** Hannah F. Warkentin, Rose Gholami Mazinan, Johannes Fuss, Leonhard Kratzer, Sarah V. Biedermann

**Affiliations:** 1 Social and Emotional Neuroscience Group, Department of Psychiatry and Psychotherapy, Center for Psychosocial Medicine, University Medical Center Hamburg-Eppendorf, Martinistraße 52, Hamburg, Germany; 2 Institute of Forensic Psychiatry and Sex Research, Center for Translational Neuro- and Behavioral Sciences, University of Duisburg-Essen, Essen, Germany; 3 Department of Psychotraumatology, Clinic St Irmingard, Osternacher Strasse 103, 83209 Prien am Chiemsee, Germany

**Keywords:** BPD, Posttraumatic stress disorder, Child sexual abuse, Emotion regulation, Sexual motivation

## Abstract

**Background:**

Increasing research has contributed to the destigmatization of sadomasochistic sexual preferences. Nevertheless, persons diagnosed with Borderline Personality Disorder (BPD) frequently report self-harmful masochistic sexual practice under the pretext of BDSM, especially those reporting experiences of child sexual abuse (CSA). Empirical research on sexual preferences in the context of BPD is scarce, although related sexual behaviors may matter particularly regarding dysfunctional and self-harming behaviors.

**Methods:**

Women with BPD (*n* = 115) and age-matched healthy controls (HC; *n* = 115) were compared regarding experiences with BDSM and masochistic fantasies, as well as associated arousal and distress. Regression and moderation analyses were conducted on cross-sectional data to examine the associations between sadomasochistic sexuality and BPD symptoms, traumatic experiences, sexual risk behavior, and sexual motivation.

**Results:**

Women with BPD practiced BDSM more often (last year: 34% vs. 15%; lifetime: 51% vs. 23%) which was associated with more autonomous, self-determined forms of sexual motivation but at the same time associated with higher BPD symptoms and risky sexuality. While a similar number of women in both groups endorsed arousal through masochistic sexual fantasies (77% vs. 74%), significantly more of those women with BPD reported associated marked distress (53% vs. 21%). Distress from masochistic fantasies was associated with less autonomous sexual motivation, in which sexuality is used in order to regulate emotions and self-esteem, and was predicted by the interaction of the severity of childhood sexual abuse and this regulation tendency.

**Conclusion:**

Sadomasochistic sexuality and corresponding fantasies in women can be an autonomous, self-determined form of sexuality. However, in women with BPD they tend to be associated with BPD symptoms, risky sexuality, problems with self-regulation and traumatization and are thus associated with marked distress. Our findings highlight the importance of considering sexual preferences in clinical context and the need for specific treatment for this subgroup suffering from their preference or acting them out in a dysfunctional or self-harming way.

**Trial registration:**

This analysis is part of a larger ongoing study and was retrospectively registered (Registration trial DRKS00029716).

**Supplementary Information:**

The online version contains supplementary material available at 10.1186/s40479-025-00283-6.

## Introduction

Sexual masochism refers to deriving sexual arousal from “being humiliated, bound, beaten, or otherwise made to suffer” (DSM-5 [1]). Since its description by Richard von Krafft-Ebing in 1886 [2] alongside sexual sadism, such preferences were long considered a pathological deviation [3]. Since the 1990s, they are encompassed under the term BDSM, an acronym for *Bondage and Discipline*,* Dominance and Submission*,* and Sadism and Masochism*, an umbrella term for sexual practices that involve consensual power exchange between individuals with specific roles divided into masochistic/submissive and sadistic/dominant [4]. After practictioners were long stigmatised [5–7], recent research has contributed to the destigmatisation, revealing that BDSM interests are prevalent in general population, with up to 47% expressing interest [8] and 40–70% of reporting BDSM-related fantasies [9–11]. Furthermore, studies indicate that BDSM practicioners generally do not exhibit more psychiatric symptoms or traumatic experiences in childhood than non-practitioners and may even display favorable psychological traits, such as higher well-being and openness to new experiences [12, 13]. However, there is evidence suggesting increased prevalences of sexual abuse in adulthood among BDSM practitioners [14].

A more differentiated view on sexual preferences in society and among clinicians has changed the perspective on sadomasochistic preferences: ICD-11 and DSM-5 now distinguish paraphilic interests or preferences from paraphilic disorders in order to differentiate between atypical sexual interests and clinically significant conditions that require intervention [15]. The diagnoses of masochistic and sadistic paraphilic disorders in the DSM-5 require considerable distress or impairment and/or the acting out with a non-consenting person [1], while the ICD-11 has introduced a category for a Coercive Sexual Sadism disorder, while sexual masochism and consensual sadomasochistic behaviors are no longer diagnosable [16].

Despite this progress, an intersection of BDSM and Borderline Personality Disorder (BPD) remains underexplored. BPD is characterized by emotion dysregulation, identity disturbance, and interpersonal instability [1], which may intersect with sadomasochistic sexuality. Kernberg named “polymorphous perverse sexual trends” as one of six suspicious symptoms for BPD as early as 1967 [17]. Research shows persons with BPD exhibit heightened sexual impulsivity and compulsive behaviors [18, 19], which may serve as mechanisms for emotion regulation. Furthermore, persons with BPD report ambivalence and negative attitudes towards sex, as well as reduced sexual satisfaction [19, 20]. Nevertheless, many aspects of sexuality have been left out in empirical research to date [18, 21], although specific sexual interests like BDSM could be of particular interest here. BDSM may provide persons with BPD a structured framework for exploring their sexuality, particularly given their struggles with identity disturbance - a core feature of the disorder [22]. Defined roles in BDSM contexts [4] may offer a sense of stability, while anxious-avoidant attachment patterns often observed in BPD have been linked to submissiveness within BDSM dynamics [14].

Initial empirical evidence supports an association between BPD and sexual masochism. Friás et al. [23] reported a tenfold higher prevalence of sexual masochism disorder among women with BPD compared to other personality disorders, highlighting trauma, particularly childhood sexual abuse (CSA), as a potential confounding factor. Similarly, Roma et al. [24] described a woman with BPD involved in a BDSM-related asphyxia practice, noting a history of CSA, substance use, self-harm, and sexual dysregulation. Self-harming behavior is common in BPD and often motivated by the need to relieve negative emotions, resolve interpersonal conflicts, or create positive feelings [1]. If inherent boundaries are crossed, the concept of sex as self-injury (SASI), as described in Swedish studies [25, 26], could be particularly relevant in the context of motives for masochistic sexuality in BPD. These findings align with our experience in therapeutic work with women with BPD, in which especially masochistic sexual practices are frequently reported as a form of dysfunctional emotion regulation and, in some cases, acted out in self-harmful or even life-threatening ways under the pretext of BDSM.

Trauma, particularly CSA, appears important regarding a potential association between BPD and masochistic sexuality. Research shows high rates in persons with BPD [27, 28]. CSA is linked to higher rates of sexual difficulties [29] and intrusive sexual fantasies involving force and elements of sadomasochism [30]. Women with BPD and sexual masochism disorder report significantly higher rates of CSA than women with BPD alone [23]. While most studies do not find an overall increase in childhood trauma among BDSM practitioners [12, 13], for those with a history of trauma, masochistic sexuality may have a function that goes beyond sexual gratification: case studies on survivors of trauma and theoretical work address sexual masochism to be either a re-enactment of experienced trauma, potentially leading to revictimization, or even a form of coping and healing [31–34] by repeating or replaying past traumatic sexual experiences as a way to master the trauma and its related emotions [35]. To summarize, masochistic preferences seem to be relevant in the context of BPD. Given the high rates of traumatization and the potential for BDSM being used as a form of self-harming behavior, we aimed to gain a general overview over the topic BDSM among women with BPD, and, based on preliminary research and clinical experiences, to explore the following hypotheses with a particular focus on masochistic fantasies:

1. Women with BPD show significantly higher prevalences of experiences with BDSM and sexual masochistic fantasies compared to the control group.
2. Women with BPD report more distress associated with their sexual masochistic fantasies compared to the control group.
3. The practice of BDSM and the distress from sexual masochistic fantasies is explained by experienced sexual abuse, sexual risk behavior and sexual motivation.

## Method

### Participants

Persons with BPD were recruited through out- and inpatient clinics at the University Medical Center Hamburg-Eppendorf (UKE) and in the region Munich, Germany. BPD had to be the primary, clinician-confirmed diagnosis for which these patients were receiving treatment at special units in the clinics. The BPD diagnosis was verified using DSM-5 criteria. Healthy control participants (HC) were recruited via flyers and internet advertisements. Inclusion criteria for the BPD group were diagnosed BPD and no current psychiatric disorders for HC to ensure a clearer comparison by minimizing potential confounding factors. Exclusion criteria were acute substance abuse, acute major or delusional depression, mania, or acute psychotic disorders for persons with BPD. Participants completed a set of questionnaires via the online survey tool Qualtrics ^®^^XM^ or via paper pencil, depending on their recruitment site, and provided written informed consent. An a priori power analysis using G*Power software 3.1 [36] determined a minimum sample size of 145 participants for a chi-square test examining the prevalence of experiences with BDSM, masochistic sexual fantasies and distress assuming at least a moderate effect, a power of 0.95, and an alpha error of α = 0.05. A total of 343 persons (male = 81, female = 260, non-binary = 2) participated in the study. Data of male and non-binary participants were excluded due to insufficient sample size. Out of 119 women with BPD left in the sample, four were excluded due to not responding to the items on the outcome variables. HC were matched based on age and gender. The final sample therefore consists of 230 women, 115 in each group. The study was approved by the ethics committees of the UKE and Ludwig-Maximillians-University Munich, and followed the guidelines of the Declaration of Helsinki (2013).

## Measures

### Masochistic fantasies (MF)

Masochistic sexual fantasies (MF) were assessed using a sexual preference subscale of the Fragebogen zum sexuellen Erleben und Verhalten (Cronbach’s α = 0.87) (FSEV) [37]. The first item measures sexual arousal from MF: “To what extent do you find it sexually arousing when your sexual partner uses force over you or oppresses you, e.g. ties you up, inflicts pain on you and you submit yourself?”. If arousal was reported, the subsequent item assessed the frequency of MF: “How often do you have these sexual desires?”. The last item collected the distress associated with these fantasies: “How much do you suffer from these sexual desires?”. All items were answered on a 5-point scale from “not at all” (1) to “a lot” (5) and “never” (1) to “very often” (5), respectively. The three items assessed MF across three dimensions: sexual fantasies, masturbation fantasies, and sexual behavior. A dichotomous variable was calculated to indicate the presence of arousal, frequency, or distress in one of the three dimensions. Based on the latter, the groups were divided into distress/no distress from MF.

### BDSM practice

Three items collected data on whether the participants had ever practiced BDSM (“I have practiced BDSM (Bondage, Discipline, Dominance & Submission, Sadism & Masochism) in my life” - yes/no), engaged in BDSM practices in the last year (“I have practiced BDSM (Bondage, Discipline, Dominance & Submission, Sadism & Masochism) in the last 12 months” - yes/no), and their role in it (“When you practiced BDSM, what role were/are you in?– active/sadistic/dominant/”top”/”dom” vs. passive/masochistic/submissive/“bottom”/“sub” vs. Switcher (dominant and submissive role)).

### Borderline personality disorder symptoms

The shortened version of the Borderline Symptom List (Cronbach’s α = 0.63 − 0.98) (BSL-23) [38] was used to assess the severity of BPD symptoms in the last seven days. The 23 items are answered on a 5-point Likert scale from “not at all” to “very strongly”. A supplementary 11-item scale assessed the extent of dysfunctional and self-harming behavior in the last week (BSL-S score).

### Posttraumatic stress disorder symptoms

The International Trauma Questionnaire (Cronbach’s α = 0.90 − 0.92) (ITQ) [39] was used to assess ICD-11 symptoms of PTSD and complex PTSD over the previous month. The 18 items are rated on a 5-point scale from “not at all” (0) to “very strong” (4). The German version exhibits good psychometric properties comparable to the English original [40].

### Trauma history

Traumatic events in childhood and adolescence were collected retrospectively using the Childhood Trauma Questionnaire (Cronbach’s α = 0.67 − 0.95) (CTQ [41]; German Version [42]). The 28-item instrument covers the subscales sexual abuse, physical abuse, emotional abuse, emotional neglect, and physical neglect. Severity is rated on a five-point Likert scale ranging from “not at all” (1) to “very often” (5). The German version is considered reliable and valid [43].

To assess sexual trauma in adulthood, we adapted the questions of the CSA subscale into adulthood (Cronbach’s α = 0.90) (Adult Sexual Abuse Questionnaire (ASAQ)) [44]. A person was considered a victim of child sexual abuse (CSA) or adult sexual abuse (ASA), if they met a cutoff score of 7. Revictimization was indicated by exceeding both cutoffs.

### Sexual motivation

The Sexual Motivation Scale (Cronbach’s α = 0.75 − 0.93) (SexMS) [45] measures six forms of reasons for engaging in sexual activities, spanning from intrinsic/autonomous over various extrinsic types of motivation to amotivation: intrinsic (engaging in sexual activities for their own sake and pleasure), integrated (engaging in sexual activities that align with other aspects of the self such as identity and values), identified (engaging in sexual activities because they are a normal and healthy part of life), introjected (engaging in sexual behaviors driven by internal pressures such a regulation of self-worth and aversive emotions), external (engaging in sexual behaviors driven by external pressures, such as rewards or punishments), and amotivated (complete absence of motivation) on a 7-point Likert scale from “does not correspond at all” (1) to “corresponds completely” (7). The scale is valid and reliable [45].

### Sexual risk survey

The Sexual Risk Survey (Cronbach’s α = 0.69) (SRS) [46] assesses risky sexual behaviors within the last six months over 23 items in five subscales: Uncommited, Risky, Impulsive, Intentional, Anal. The English questionnaire was translated into German and verified through backward-forward-translation before [44].

### Data analysis

Sociodemographic and clinical sample characteristics were described using descriptive statistics. For binary variables, frequencies and percentages are presented; for continuous variables, mean (*M*) and standard deviation (*SD*) are shown. Group differences were tested using Student’s t-tests and chi-square tests for women with BPD versus HC and subgroups of women with BPD based on BDSM practice or distress from sexual fantasies. Two-tailed p-values are given for differences in demographic data, whereas one-tailed p-values for variables related to hypothesis testing. Associations between variables were tested by Pearson or point-biserial correlation analyses. As a replication of the results of Frías et al. [23], multivariate analyses of variance (MANOVA) were conducted to examine differences in childhood trauma between the groups BDSM/no BDSM and distress from MF/no distress from MF in women with BPD. Similarly, the groups were compared regarding their sexual motivation. A binary logistic regression analysis with the independent variables CTQ CSA, BSL, BSL-S, and SRS was performed to predict BDSM experience in the last 12 months. Multiple linear regression analyses were conducted to examine the influence of the independent variables CTQ CSA, BSL, BSL-S, and SRS on the distress from MF. Age was used as control variable. Data was checked regarding the assumptions for calculating a multiple linear regression using various analyses.

Finally, in order to test whether the distress associated with the MF in the three dimensions sexual fantasies, masturbation fantasies, and sexual behavior is predicted by the simultaneous presence of CSA and introjected sexual motivation (SM-Introj), a sexual motivation relating to self-worth and emotion regulation that could therefore be of particular interest in the context of BPD, moderator analyses were conducted. After the exclusion of two outliers, the predictor variables, CSA and SM-Introj, were mean centered. Age was used as control variable. In a hierarchical approach, the predictors were first entered individually and then the interaction of both predictors (CSA*SM-Introj) was included in the models. Effect sizes were calculated for the different analyses: *d* for Student’s t-tests, *η²* for MANOVA, φ for chi-square tests, Odd’s ratio for the logistic regression and Cohen’s *f*² for the multiple regression analyses. To assess potential multicollinearity, the respective independent variables of the regression analyses were correlated with each other (see supplements). Missing data on individual items concerning experiences with (sexual) abuse or masochistic fantasies were likely not missing at random, given the sensitive nature of these topics. Instead of imputing these values, the missing data was marked in the relevant tables. The analyses were conducted using IBM SPSS Statistics Version 25.0 [47] and SPSS Macro PROCESS (v3.5) in an explorative manner without adjustment for multiplicity. Statistical significance was determined by 95% confidence intervals.

## Results

### Sample characteristics

The final sample consists of 115 women diagnosed with BPD and 115 HC matched by age. Women with BPD had a higher probability of being single, not highly educated, not working full-time, and non-heterosexual. The proportion of women with PTSD or cPTSD, as well as those having been sexually abused in childhood, adulthood, or both, was significantly higher in the BPD group, see Table 1.

**Table 1:** Sociodemographic characteristics in women with and without BPD

	BPD (*n* = 115)		HC (*n* = 115)		Statistic			Effect size
	*M*	*SD*	*M*	*SD*	*U*		*p*	
age (years)	*28.65*	*7.80*	*28.99*	*7.46*	*6*,*950.00*		0.503	*η²*=0.00
	*n*	*%*	*n*	*%*	df	*χ* ^*2*^	p	
marital status, single	61	53.1	39	33.9	1	8.56	0.005	φ = 0.19
children, yes	14	12.2	15	13.0	1	0.04	1	φ=-0.01
school education, high	49	42.6	88	76.5	1	27.46	< 0.001	φ = 0.35
occupation, full-time	19	16.5	39	33.9	1	9.22	0.004	φ = 0.20
heterosexual orientation	39	33.9	65	56.5	1	11.87	0.001	φ = 0.23
comorbid trauma diagnosis								
PTSD	55	47.8	-	-	1	72.29	< 0.001	φ=-0.56
cPTSD	50	43.5	-	-	1	63.89	< 0.001	φ=-0.53
experienced sexual abuse								
CSA	68^a^	60.7	10	8.7	1	68.07	< 0.001	φ=-0.55
ASA	52^b^	49.1	10	8.7	1	44.52	< 0.001	φ=-0.45
revictimization	42^c^	40.8	3	2.6	1	48.32	< 0.001	φ=-0.47
BDSM role (masoc.)	38^e^	70.4	13	50.0	1	3.15	0.064	φ=-0.20
Aroused by MF1	83	72.2	81	70.4	1	0.09	0.442	φ=-0.02
Frequency of MF1	80^f^	89.9	70	82.4	1	2.08	0.111	φ=-0.11
Distress from MF	38^f^	42.7	11	12.9	1	19.03	< 0.001	φ=-0.33
Aroused by MF2	71	62.3	73	63.5	1	0.04	0.480	φ = 0.01
Frequency of MF2	70^f^	78.7	68	80.0	1	0.05	0.487	φ = 0.02
Distress from MF2	33^f^	37.5	10	11.8	1	15.33	< 0.001	φ=-0.30
Aroused by MF3	76	66.1	70	60.9	1	0.68	0.247	φ=-0.05
Frequency of MF3	75^f^	84.3	64	75.3	1	2.18	0.099	φ=-0.11
Distress from MF3	38^f^	42.7	15	17.6	1	12.88	< 0.001	φ=-0.27

Note. BPD = Borderline Personality Disorder, HC = Healthy Controls, (c)PTSD = (complex) Posttraumatic Stress Disorder, CSA = Child Sexual Abuse, ASA = Adult Sexual Abuse, BDSM = Sexual activities related to Bondage & Discipline, Dominance & Submission, Sadism & Masochism, MF = masochistic fantasies, MF1 = MF in sexual fantasies, MF2 = MF in masturbation fantasies, MF3 = MF in sexual behavior. a: missing patient data *n* = 3. b: missing patient data *n* = 9. c: missing patient data *n* = 12, d: missing patient data *n* = 9. e: percentages relate to women having practiced BDSM. f: percentages relate to women having MF

### BDSM experiences and masochistic fantasies (MF) in women with and without BPD

**Fig. 1:**
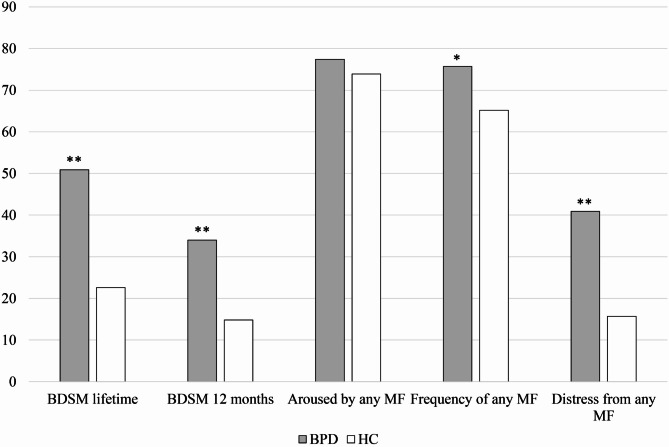
Experiences with BDSM and masochistic fantasies in women with and without BPD in % ***Note***. BPD = Borderline Personality Disorder, HC = Healthy Controls, BDSM = Sexual activities related to Bondage & Discipline, Dominance & Submission, Sadism & Masochism, BDSM lifetime/12 months: missing patient data *n* = 9; MF = masochistic fantasies. * indicates statistical significance with *p* <.05; ** indicates statistical significance with *p* <.001

Women with BPD reported significantly more BDSM experiences, both within the last year (34% vs. 15%, *χ*^*2*^ = 11.13, *p* =.001, φ = -0.22) and over their lifetime (51% vs. 23%, *χ*^*2*^ = 19.18, *p* <.001, φ = -0.30; see Fig. 1). They were more likely to take on a masochistic role on trend level (Table 1). While there were no significant differences between women with BPD and HC in terms of arousal or frequency of MF in all three dimensions, more women with BPD reported distress from MF in all dimensions, see Table 1. Overall, there were no significant differences between the groups in the arousal from MF (77% vs. 74%, *χ*^*2*^ = 0.38, *p* =.323, φ = -0.04), but the frequency of having any MF was higher in the BPD group (76% vs. 65%, *χ*^*2*^ = 6.13, *p* =.013, φ = -0.19), see Fig. 1. As with the single dimensions, women in the BPD group experienced significantly more distress from any MF than women in the HC group (40% vs. 16%, *χ*^*2*^ = 19.59, *p* <.001, φ = -0.33).

### Associations between BDSM experiences and masochistic sexual fantasies

In the BPD group, BDSM 12 months experience was significantly correlated with being aroused by MF in sexual fantasies (*r* =.407, *p* <.001), in masturbation fantasies (*r* =.382, *p* <.001), and in sexual behavior (*r* =.503, *p* <.001) as well as the frequency of these fantasies (MF in SF: *r* =.258, *p* =.019; in MF: *r* =.228, *p* =.038; in SB: *r* =.491, *p* <.001). Lifetime BDSM experiences were also associated with arousal in sexual fantasies (*r* =.376, *p* <.001), masturbation fantasies (*r* =.368, *p* <.001), and sexual behavior (*r* =.365, *p* <.001) as well as the frequency of these fantasies (MF in SF: *r* =.429, *p* <.001; in MF: *r* =.337, *p* =.002; in SB: *r* =.410, *p* <.001). Results concerning the HC show similar correlations between having experienced BDSM and arousal (last 12 months: MF in SF: *r* =.306, *p* =.001; in MF: *r* =.386, *p* <.001; in SB: *r* =.379, *p* <.001; lifetime: MF in SF: *r* =.398, *p* <.001; in MF: *r* =.473, *p* <.001; in SB: *r* =.487, *p* <.001) as well as the frequency of these fantasies (last 12 months: MF in SF: *r* =.246, *p* =.023; in MF: *r* =.318, *p* =.003; in SB: *r* =.293, *p* =.006; lifetime: MF in SF: *r* =.432, *p* <.001; in MF: *r* =.495, *p* <.001; in SB: *r* =.455, *p* <.001). No significant associations between BDSM experiences and distress from MF were found in either group.

### BDSM experiences

#### Prevalence of BDSM, BPD symptoms and sexual risk behavior

Regarding BPD symptoms, a weak but significant correlation was found between the 12 months BDSM prevalence and the BSL-S score in the BPD group (*r* =.283, *p* =.003). No significant associations were found in HC. While the lifetime BDSM prevalence did not show any significant associations to sexual risk behavior in the BPD group, the 12 months prevalence was significantly correlated to all subscales except SRS Impulsive: SRS Uncommitted (*r* =.204, *p* =.036), SRS Risky (*r* =.267, *p* =.006), SRS Intentional (*r* =.235, *p* =.016) and SRS Anal (*r* =.197, *p* =.043) as well as the overall SRS score (*r* =.324, *p* =.001). In HC, the 12 months BDSM prevalence was significantly correlated with SRS Uncommitted (*r* =.205, *p* =.035), while the lifetime prevalence was significantly associated with SRS Impulsive (*r* =.206, *p* =.034) and SRS Anal (*r* =.215, *p* =.027).

#### Lifetime prevalence of BDSM, PTSD and sexual abuse in women with BPD

Comparing women with BPD with and without BDSM experiences in their lifetime there were no significant differences regarding comorbid (c)PTSD diagnoses, nor in the prevalence and severity of exposure to CSA, but in the prevalence and severity of exposure to ASA and the prevalence of revictimization which was significantly higher in the BDSM group.

**Table 2:** Trauma diagnoses and sexual abuse in women with BPD with and without BDSM experiences in their lifetime

	BPD (*n* = 106)		Difference			Effect size
	BDSM lifetime (*n* = 54)	No BDSM lifetime (*n* = 52)	df	Statistic	*p*	
PTSD (*n* (%))	30 (55.6)	22 (42.3)	1	*χ*^*2*^ = 1.86	0.121	φ = 0.13
cPTSD (*n* (%)	27 (50.0)	20 (38.5)	1	*χ*^*2*^ = 1.43	0.159	φ = 0.12
CSA (*n* (%))	33 (63.5)^a^	31 (60.8)^b^	1	*χ*^*2*^ = 0.08	0.469	φ = 0.03
CSA score (*M* (*SD*))	2.83 (1.18)^a^	2.73 (1.25)^b^	-	*U* = 1,380.00	0.709	*η²*=0.00
ASA (*n* (%))	35 (64.8)^a^	17 (32.7)^a^	1	*χ*^*2*^ = 10.94	0.001	φ = 0.32
ASA score (*M* (*SD*))	2.56 (0.97)^c^	1.92 (1.05)^a^	-	*U* = 1,615.50	0.002	*η²*=0.10
Revictim (*n* (%))	27 (51.9)^a^	15 (29.4)^b^	1	*χ*^*2*^ = 5.40	0.017	φ = 0.23

Note. BPD = Borderline personality disorder, BDSM = Sexual activities related to Bondage & Discipline, Dominance & Submission, Sadism & Masochism (c)PTSD = (complex) Posttraumatic Stress Disorder, CSA = Child Sexual Abuse, ASA = Adult Sexual Abuse, Revictim = Revictimization; a: missing patient data *n* = 2. b: missing patient data *n* = 1. c: missing patient data *n* = 6

#### Lifetime prevalence of BDSM and childhood trauma in women with BPD

There were no significant differences in any of the CTQ subscales when comparing persons with BPD with and without BDSM lifetime experiences (*F* = 0.00 to 0.14, *p* =.707 to 0.990).

#### 12-months-prevalence of BDSM and sexual motivation in women with BPD

Regarding sexual motivation measured by SexMS, a MANOVA showed that women with BPD who had practiced BDSM in the last 12 months scored significantly higher on the Intrinsic (*F*(6,99) = 8.09, *p* =.005), Integrated (*F*(6,99) = 8.86, *p* =.004), Identified (*F*(6,99) = 13.44, *p* <.001) subscales, and significantly lower on the Amotivation scale (*F*(6,99) = 4.06, *p* =.046). No significant differences were found in the Introjected and External subscales (*F* = 1.66 and 1.21, *p* =.200 and 0.274).

#### Prediction of 12-months-prevalence of BDSM in women with BPD

The binary logistic regression model predicting BDSM experiences in the last 12 months among women with BPD was significant, *χ*^*2*^ = 25.29 (*p* <.001), resulting in a small to medium amount of explained variance (Backhaus et al., 2006), as shown by Nagelkerke’s *R*² = 0.30. Significant predictors were BSL-S score (*p* =.005) and SRS score (*p* =.026).

**Table 3:** Binary logistic regression predicting BDSM 12 months prevalence

	B	SE	Wald	*p*	Odds Ratio	95% CI for Odds Ratio	
						LL	UL
CSA	-0.02	0.22	0.01	0.946	0.99	0.65	1.50
BSL	-0.01	0.01	0.86	0.354	0.35	0.96	1.01
BSL-S	0.21	0.07	7.62	0.006	1.23	1.06	1.42
SRS	0.00	0.00	5.43	0.020	1.00	1.00	1.01
age	-0.05	0.03	1.86	0.173	0.96	0.90	1.02
Constant	-0.14	1.08	0.02	0.897	0.87		

Note. *N* = 103. CSA = Child sexual abuse, BSL = Borderline Symptom List sum score, BSL-S = Borderline Symptom List Dysfunctional Behavior Scale, SRS = Sexual Risk Scale

### Masochistic fantasies

#### Distress, trauma, arousal and frequency of MF in women with BPD

Women with BPD who reported distress associated with their masochistic fantasies showed significantly higher prevalences of (c)PTSD diagnoses and higher CSA scores, reflecting the severity of exposure to CSA. There were no significant differences regarding the other subscores of CTQ (see supplements). Women with BPD reporting distress were more likely to be victims of revictimization and experienced these fantasies significantly more often (except in masturbation fantasies). They also reported significantly higher arousal from these fantasies across all three dimensions examined, see Table 4.

**Table 4:** Clinical characteristics in women with BPD with and without distress from masochistic fantasies

	BPD with masochistic fantasies (*n* = 89)		Difference			Effect size
	Distress (*n* = 47)	No distress (*n* = 42)	df	Statistic	*p*	
PTSD (*n* (%))	29 (61.7)	16 (38.1)	1	*χ*^*2*^ = 4.95	0.022	φ = 0.24
cPTSD (%)	28 (59.6)	15 (35.7)	1	*χ*^*2*^ = 5.06	0.021	φ = 0.24
CSA (*n* (%))	33 (73.3)^a^	17 (41.5)^b^	1	*χ*^*2*^ = 8.95	0.003	φ = 0.32
CSA score (*M* (*SD*))	3.13 (1.06)^a^	2.24 (1.24)^b^	-	*U* = 1,289.00	0.001	*η²*=0.13
ASA (*n* (%))	25 (55.6)^a^	16 (42.1)^c^	1	*χ*^*2*^ = 1.49	0.158	φ = 0.13
ASA score (*M* (*SD*))	2.44 (1.10)^d^	2.17 (1.01)^e^	-	*U* = 817.50	0.280	*η²*=0.02
Revictimization (*n* (%))	23 (53.5)^c^	11 (29.7)^d^	1	*χ*^*2*^ = 4.59	0.027	φ = 0.24
Aroused by MF1 (*M* (*SD*))	4.00 (1.22)	3.31 (1.35)	-	*U* = 1,271.50	0.015	*η²*=0.07
Frequency of MF1 (*M* (*SD*))	3.96 (1.30)	3.21 (1.51)	-	*U* = 1,265.00	0.017	*η²*=0.06
Aroused by MF2 (*M* (*SD*))	3.50 (1.44)^b^	2.81 (1.55)	-	*U* = 1,216.50	0.032	*η²*=0.05
Frequency of MF2 (*M* (*SD*))	3.34 (1.49)	2.79 (1.60)	-	*U* = 1,193.00	0.082	*η²*=0.03
Aroused by MF3 (*M* (*SD*))	3.57 (1.46)	2.93 (1.31)	-	*U* = 1,252.50	0.025	*η²*=0.06
Frequency of MF3 (*M* (*SD*))	3.49 (1.50)	2.79 (1.39)	-	*U* = 1,258.00	0.022	*η²*=0.06

Note. BPD = Borderline personality disorder, (c)PTSD = (complex) Posttraumatic Stress Disorder, CSA = Child Sexual Abuse, MF = masochistic fantasies, MF1 = MF in sexual fantasies, MF2 = MF in masturbation fantasies, MF3 = MF in sexual behavior, a: missing patient data *n* = 2, b: missing patient data *n* = 1, c: missing patient data *n* = 4, d: missing patient data *n* = 6, e: missing patient data *n* = 7, d: missing patient data *n* = 5

#### Distress associated with MF and sexual motivation in women with BPD

Regarding sexual motivation, MANOVA showed that women with BPD reporting distress associated with their MF scored significantly higher on the SM-Introj subscale (*F*(6,82) = 11.35, *p* =.001, Wilks λ = 0.86). The other subscales of the SexMS did not yield between-group differences, see Table 5.

**Table 5:** Sexual motivation in women with BPD with and without distress from masochistic fantasies

	BPD with masochistic fantasies (*n* = 89)		Difference		Effect size
Variables	Distress (*n* = 47)	No distress (*n* = 42)	Statistic	*p*	*η²*
*SexMS*					
Intrinsic	3.27 (1.75)	3.42 (1.63)	*F* = 0.18	0.677	0.002
Integrated	2.53 (1.81)	2.22 (1.79)	*F* = 0.66	0.417	0.008
Identif	2.81 (1.48)	2.64 (1.43)	*F* = 0.31	0.582	0.004
Introj	3.06 (1.32)	2.14 (1.26)	*F* = 11.35	0.001	0.115
Extern	2.69 (1.72)	2.24 (1.80)	*F* = 1.44	0.234	0.016
Amotivation	1.44 (1.55)	1.23 (1.40)	*F* = 1.01	0.318	0.011

Note. BPD = Borderline personality disorder, SexMS = Sexual Motivation Scale. Values range between 0 and 6

#### Prediction of distress associated with masochistic fantasies in women with BPD

Multiple linear regression analyses were performed to predict the distress associated with MF in women with BPD. The first model significantly explained 34% of the variance in distress associated with MF in sexual fantasies (*p* <.001) with CSA (*ß* = 0.30, *p* =.014), BSL (*ß* = 0.31, *p* =.014), and SM-Introj (*ß* = 0.24, *p* =.016) being significant predictors. The second model, predicting MF in masturbation fantasies, explained 20% of the variance (Cohen’s *f*^*2*^ = 0.25, *p* =.013). CSA (*ß* = 0.26, *p* =.045) was the only significant predictor. For MF in sexual behavior, the third model predicted 21% of the variance in the outcome (Cohen’s *f*^*2*^ = 0.27, *p* =.007). CSA (*ß* = 0.30, *p* =.023) and SM-Introj (*ß* = 0.30, *p* <.006) were significant predictors.

**Table 6:** Multiple linear regression analyses predicting distress from masochistic fantasies in sexual fantasies, masochistic fantasies, and sexual behavior

	Model 1 (MF in SF; *n* = 79)				Model 2 (MF in MF; *n* = 78)				Model 3 (MF in SB; *n* = 79)			
	*B*	95% CI for B		*ß*	*B*	95% CI for B		*ß*	*B*	95% CI for B		*ß*
		*LL*	*UL*			*LL*	*UL*			*LL*	*UL*	
Constant	0.23	-0.79	1.24		0.83	-0.26	1.91		0.91	-0.22	2.03	
CSA	0.28	0.06	0.50	0.30^*^	0.24	0.01	0.47	0.26^*^	0.28	0.04	0.52	0.30^*^
BSL	0.02	0.00	0.03	0.31^*^	0.01	-0.00	0.03	0.21	0.00	-0.01	0.02	0.07
BSL-S	− 0.03	-0.09	0.04	− 0.09	− 0.03	-0.10	0.05	− 0.09	− 0.02	-0.09	0.06	− 0.06
SRS	0.00	-0.00	0.00	− 0.08	0.00	-0.00	0.00	− 0.06	0.00	-0.00	0.00	− 0.03
SM-Introj	0.21	0.04	0.38	0.24^*^	0.15	-0.03	0.33	0.18	0.27	0.08	0.45	0.30^*^
age	− 0.01	-0.04	0.02	− 0.08	− 0.02	-0.05	0.01	− 0.13	− 0.02	-0.06	0.01	− 0.15

Note. CSA = Child Sexual Abuse (CTQ), BSL-23 = Borderline Symptom List 23, SRS = Sexual Risk Scale. ^*^p <.05 ^**^p <.001

Moderation analyses were conducted to test whether the interaction between severity of CSA and SM-Introj significantly predicts the distress from MF. In the first model, both CSA and SM-Introj showed to be significant predictors of distress from MF in sexual fantasies in the first step, (*F*(3, 80) = 10.73, *p* <.001), accouting for 29% of the variance (Cohen’s *f*^*2*^ = 0.41). Adding the interaction term of the two independent variables in a second step, the model also was significant, (*F*(4, 79) = 9.90, *p* <.001), explaining 33% of the variance (Cohen’s *f*^*2*^ = 0.49). The severity of CSA significantly moderated the effect between SM-Introj on distress from MF (*ß* = 0.22, *p* =.021, ∆*R*^2^ = 0.05).

The second model, predicting distress from MF in masturbation fantasies, showed similar results: CSA and SM-Introj significantly predicted the outcome in the first step, *F*(3, 79) = 5.85, *p* =.001, explaining 18% of the variance (Cohen’s *f*^*2*^ = 0.22), with their interaction also being a significant predictor in the second hierarchical step, (*F*(4, 78) = 5.77, *p* <.001, *ß* = 0.22, *p* =.010), explaining an additional 5% (Cohen’s *f*^*2*^ = 0.30).

The third and final moderation model also showed CSA and SM-Introj to be significant predictors of the distress from MF in sexual behavior, *F*(3, 80) = 10.39, *p* <.001, with 28% explained variance in the first step (Cohen’s *f*^*2*^ = 0.39). After the entering of the interaction of both variables, the model remained significant, *F*(4, 79) = 10.29, *p* <.001, *ß* = 0.25, *p* =.008, with an additional 6% of variance being explained in this second step (Cohen’s *f*^*2*^ = 0.52).

**Table 7:** Moderation analyses to predict distress from MF in women with BPD

Distress from MF in sexual fantasies (*n* = 83)							
	*B*	95% CI for B		*SE B*	ß	*R* ^*2*^	*ΔR* ^*2*^
		*LL*	*UL*				
Step 1						0.29	0.29^**^
Constant	2.01	1.26	2.76	0.38			
CSA	0.34	0.18	0.50	0.08	0.40^**^		
SM-Introj	0.24	0.09	0.38	0.07	0.31^*^		
age	-0.01	-0.04	0.01	0.01	− 0.08		
Step 2						0.33	0.05^**^
Constant	2.02	1.29	2.75	0.37			
CSA	0.33	0.17	0.49	0.08	0.39^**^		
SM-Introj	0.24	0.09	0.37	0.07	0.31^*^		
age	-0.01	-0.04	0.01	0.01	− 0.09		
SM * CSA	0.13	0.02	0.24	0.06	0.22^*^		
Distress from MF in masturbation fantasies (*n* = 82)							
	*B*	95% CI for B		*SE B*	ß	*R* ^*2*^	*ΔR* ^*2*^
		*LL*	*UL*				
Step 1						0.18	0.18^**^
Constant	2.10	1.34	2.86	0.38			
CSA	0.25	0.08	0.41	0.08	0.31^*^		
SM-Introj	0.18	0.03	0.33	0.07	0.25^*^		
age	-0.02	-0.04	0.01	0.01	− 0.14		
Step 2						0.23	0.05^**^
Constant	2.11	1.36	2.85	0.37			
CSA	0.23	0.07	0.39	0.08	0.29^*^		
SM-Introj	0.18	0.04	0.33	0.07	0.25^*^		
age	-0.02	-0.05	0.01	0.01	− 0.15		
SM * CSA	0.12	0.01	0.23	0.06	0.22^*^		
Distress from MF in sexual behavior (*n* = 83)							
	*B*	95% CI for B		*SE B*	ß	*R* ^*2*^	*ΔR* ^*2*^
		*LL*	*UL*				
Step 1						0.28	0.28^**^
Constant	2.39	1.63	3.15	0.38			
CSA	0.22	0.05	0.38	0.08	0.25^*^		
SM-Introj	0.33	0.18	0.48	0.08	0.42^**^		
age	-0.03	-0.05	0.00	0.01	− 0.19		
Step 2						0.34	0.06^**^
Constant	2.40	1.66	3.13	0.37			
CSA	0.20	0.04	0.36	0.08	0.23^**^		
SM-Introj	0.33	0.18	0.47	0.07	0.42^**^		
age	-0.03	-0.00	-0.00	0.01	− 0.20		
SM * CSA	0.15	0.26	0.26	0.06	0.25^*^		

Note. CI = confidence interval; LL = lower limit; UL = upper limit; SM-Introj = Introjected sexual motivation (SexMS); CSA = Child sexual abuse score (CTQ). ^*^p <.05 ^**^p <.001

## Discussion

This paper aimed at exploring associations between sadomasochistic sexuality and BPD. Thereby, this study addresses the paucity of empirical research on BPD and sexuality in general, and BPD and specific sexual preferences in particular, which has already been stated before [18, 21, 44].

Our study shows that masochistic sexual fantasies are equally frequent in healthy women and women with BPD and that a similar number of women in both groups report experiencing arousal from these fantasies. However, a higher proportion of women in the BPD group compared to the control group reported engaging in BDSM-related sexual activities both throughout their lifetime and in the last year, with a greater tendency to take on the masochistic part. The practice of BDSM within the last year was associated with the extent of dysfunctional and self-harming behavior as well as risky sexual behavior. The key differentiator regarding the masochistic sexual fantasies is the prevalence of distress associated with these fantasies, which was significantly higher in the BPD sample. The severity of this distress was mainly predicted by BPD symptomatology, experiences of CSA, and introjected sexual motivation. Moreover, the effect of CSA on distress was moderated by the level of emotion and self-worth regulation through sexuality.

### Prevalences of experiences with BDSM and sexual masochistic fantasies

Our finding that most of the women in both groups endorsed arousal from masochistic sexual fantasies and reported a similar frequency of these fantasies aligns with preliminary studies showing that interest in and fantasies about BDSM-related practices are highly prevalent in the general population [4, 8–11], while only a smaller proportion actually engages in BDSM-related activities [9].

We were able to confirm the hypothesis that women with BPD are more likely to engage in sexual activities related to BDSM than women without BPD. The prevalence in the BPD group was twice as high as in the control group over their lifetime and even three times as high over the last twelve months prevalence. In line with the results by Frías et al. [23] reporting an association between BPD and masochistic sexuality, we found a higher tendency to take on the masochistic part in BDSM-related practices.

In our analyses, we found a positive association between engaging in BDSM practices and both arousal from and frequency of masochistic fantasies, suggesting that women with such fantasies may be more inclined to incorporate them into their sexual behavior. Interestingly, this association was stronger in the BPD group. Moreover, women with BPD who reported having masochistic fantasies also experienced greater distress associated with them. This finding of heightened sexual arousal alongside higher distress seems contradictory at first. So far, previous research has shown that persons with BPD often have more ambivalence and stronger negative attitudes towards sex and report lower sexual satisfaction [19, 20]. These seemingly contradictory findings can be better understood when considering the results of the subsequent analyses. These initial results already underscore that there is a diverse and multifaceted nature of BDSM-related experiences in persons with BPD: for some, fantasies and sexual behaviors are closely intertwined, while for others, these experiences may provoke significant distress, and for some, both aspects may coexist.

### BDSM experiences in women with BPD

Our finding that women with BPD who have experienced BDSM-related practices in their lifetime do not score higher on (c)PTSD or childhood maltreatment is consistent with studies reporting BDSM not to be associated to traumatic events or psychiatric symptoms [12, 13]. Additionaly, those women with BPD who reported having practiced BDSM in the last year showed higher scores in intrinsic, as well as integrated, and identified sexual motivation, which are considered more autonomous forms of motivation. This suggests again that for some women, engaging in BDSM practices is part of an autonomous, self-determined and maybe also conscious decision to explore and express their sexual preferences. Given that the sexuality of women with BPD is often portrayed as compulsive and externally driven, these findings point to the potential of sexuality as a psychological resource. Further research into the adaptive and enriching aspects of BDSM-related practices is essential to provide a more nuanced understanding of sexual expression in this population.

On the other hand, those women with BPD who previously have engaged in BDSM showed a higher prevalence of ASA and revictimization, meaning they were more likely to be sexually abused in adulthood or both, in childhood and adulthood. An increased prevalence of sexual abuse in adulthood among BDSM practitioners has been reported before [14]. Moreover, the already known tendency towards risky sexual behavior and unsafe sexual practices [18, 19, 48, 49] seems to matter regarding BDSM practices as well: the practice of BDSM in the last year was predicted by dysfunctional behavior within the context of BPD symptomatology and sexual risk behavior. Although it cannot be derived from our data if this increased sexual abuse in adulthood was experienced while practicing BDSM, this should be considered in further research as well as in clinical work with BPD patients reporting such experiences [23].

### Distress associated with sexual masochistic fantasies in women with BPD

In the subgroup of persons with BPD reporting distress associated with their masochistic sexual fantasies, the higher prevalence of (c)PTSD and CSA suggests that this distress might be associated to traumatization. We could replicate the finding of Frías et al. [23] that CSA is the only differentiating form of childhood abuse between the groups. Additionally, women reporting distress endorsed higher arousal and frequency of these fantasies. These findings align with the review by Gewirtz-Meydan and Opuda [30], which found that CSA survivors were more likely to report sexual fantasies involving force and elements of sadomasochism. Also, a higher experience of shame and guilt regarding sexuality is reported in this group [50]. The distress in our sample was explained by CSA, BPD symptoms, and introjected sexual motivation, which was also generally higher in the group of women reporting distress from their fantasies. Moreover, moderation analyses indicated that distress related to CSA was particularly elevated in women showing higher introjected sexual motivation. In this context, the preoccupation with such distressing fantasies could be seen as dysfunctional behavior or a form of sexual self-injury (SASI) to cope with aversive states of tension resulting from emotional and self-esteem dysregulation, as previously reported in persons with BPD [25, 26, 51]. Although complex to disentangle, these findings suggest that not only the traumatization and its consequences are decisive for the distress, but also specific BPD characteristics. It is plausible that for some women with BPD and CSA experiences, masochistic sexual fantasies may serve as either a form of intrusive re-experiencing during sexual activity or an active attempt to cope with the trauma and its consequences [31–33].

### Limitations

The present study has some limitations. Firstly, the inclusion of only female participants limits the generalizability of our findings. Similarly, the exclusion of specific comorbid disorders also narrows the scope of the results.Then, our study is bounded by the limitations of correlational design due to the cross-sectional character of the data. Thus, claims regarding the temporal relations between variables cannot be made.

The measure used to assess masochistic sexual fantasies consists of only three items derived from a sexual preferences subscale. As there are no validated questionnaires to date, we chose to use this one because it had already been developed and applied in previous studies.

Moreover, although CSA was a central focus of our analyses, we have not yet considered PTSD symptoms as predictor due to the small sample size of women with BPD and a comorbid PTSD diagnosis. Here, we focused on BPD symptomatology. Future research should investigate the influence of PTSD symptoms not only in persons with BPD but also in persons with PTSD without comorbid BPD, aiming to disentangle the effects of both disorders on this topic as far as possible. This would also be useful to understand to what extent sexual fantasies causing suffering occur in the form of specific PTSD symptoms like intrusive re-experiencing.

The BSL-S score only measures the extent of dysfunctional behavior in the last week. Further research should consider assessing this construct over a longer period, particularly when simultaneously interpreting prevalences over a year or across the lifespan.

Lastly, the instrument collecting masochistic fantasies firstly assesses arousal from the fantasies before measuring their frequency and resulting distress. If no arousal was reported, the frequency and distress was not assessed. It can certainly be assumed that there are also women who have fantasies but experience them exclusively as aversive and not at all as erotic. Unfortunately, these women could not be represented in our results.

## Conclusion

We conclude that addressing sadomasochistic sexuality and fantasies is important in clinical and scientific work with patients diagnosed with BPD. The positive association between BDSM experiences and autonomous sexual motivations should stimulate further research into the adaptive and enriching aspects of BDSM-related practices to provide a more nuanced understanding of sexual expression in this population. On the other hand, an association between the engagement in BDSM practices in the last year and risky and dysfunctional behaviors in women with BPD was found. The distress, which alongside impairment, is crucial in transforming symptoms into potential disorders, is notably present in women who have experienced childhood sexual abuse and who are motivated to engage in sexual activities to reduce internal pressures. Our findings suggest that sexual masochism, though no longer diagnosed as an independent pathological condition, could be part of the specific symptomatology of BPD in terms of self-regulation and dysfunctional behaviors.

These associations require further empirical investigation. Clinically and therapeutically, our results highlight the importance of addressing sexuality within this group. It is essential to approach the wide range of motives for sexual preferences with sensitivity and without stigmatization. Only a subset of persons with BPD will need specialized treatment for self-harming or dysfunctional BDSM practices and will likely benefit from work on emotion regulation and self-worth. Integrative treatment concepts that include specific sexual therapy interventions could be promising. Engaging in sadomasochistic sexuality or having related fantasies is not necessarily linked to mental illness or distress; for some women, it may be an autonomous choice and a potential resource.

## Electronic supplementary material

Below is the link to the electronic supplementary material.

Supplementary Material 1

Supplementary Material 2

## Data Availability

No datasets were generated or analysed during the current study.

## Abbreviations

ASA: Adult Sexual Abuse
ASAQ: Adult Sexual Abuse Questionnaire
BDSM: Bondage and Discipline, Dominance and Submission, and Sadism and Masochism
BPD: Borderline Personality Disorder
BSL: Borderline Symptoms in the last week
BSL-23: Borderline Symptom List 23
BSL-S: Dysfunctional and self-harming behavior in the last week
(c)PTSD: (Complex) Posttraumatic stress disorder
CSA: Child sexual abuse
CTQ: Childhood Trauma Questionnaire
DSM-5: Diagnostic and Statistical Manual of Mental Disorders 5
HC: Healthy controls
ICD-11: International Classification of Diseases 11th Version
ITQ: International Trauma Questionnaire
M: Mean
MANOVA: Multivariate Analysis of Variance
MF: Masochistic sexual fantasies
SD: Standard Deviation
SexMS: Sexual Motivation Scale
SM-Introj: Introjected Sexual Motivation
SRS: Sexual Risk Survey
UKE: University Medical Center Hamburg-Eppendorf
