# Supplementary material for: BDSM and masochistic sexual fantasies in women with borderline personality disorder: simply on the spectrum of “normality” or source of suffering?
Source: Borderline Personal Disord Emot Dysregul. 2025 Feb 22;12:7. doi: 10.1186/s40479-025-00283-6 (PMC11847330; doi:10.1186/s40479-025-00283-6)
Supplement: Supplementary file 1 — Supplementary Material 1 [file 40479_2025_283_MOESM1_ESM.docx]

**Supplements**

We here present results of a MANOVA, replicating the results of Frías et al. (2017).

**Table Supplements 1.**

Childhood trauma in women with BPD with masochistic fantasies with and without suffering from these.

|  | BPD with  masochistic fantasies (*n*=84) | | Difference | | Effect size |
| --- | --- | --- | --- | --- | --- |
| Variables | Suffering (*n*=47) | Not suffering (*n*=42) | Statistic | *p* | *η²* |
| *CTQ* |  |  |  |  |  |
| Emotional abuse | 3.34 (1.05) | 3.44 (0.87) ^a^ | *F*=0.37 | .547 | .004 |
| Physical abuse | 2.17 (1.17) | 1.81 (1.15) | *F*=2.22 | .140 | .026 |
| Sexual abuse | 3.13 (1.06) ^b^ | 2.24 (1.24) ^a^ | *F*=12.40 | .001 | .131 |
| Emotional neglect | 2.94 (1.17) | 3.00 (1.15) | *F*=0.12 | .735 | .001 |
| Physical neglect | 2.63 (1.22) ^a^ | 2.33 (1.20) | *F*=1.84 | .179 | .022 |

***Note***. BPD = Borderline personality disorder, CTQ = Child trauma questionnaire. Values range between 5 and 35. a: missing patient data n = 1. b: missing patient data n = 2.
