# Supplementary material for: BDSM and masochistic sexual fantasies in women with borderline personality disorder: simply on the spectrum of “normality” or source of suffering?
Source: Borderline Personal Disord Emot Dysregul. 2025 Feb 22;12:7. doi: 10.1186/s40479-025-00283-6 (PMC11847330; doi:10.1186/s40479-025-00283-6)

**Supplements**

We here present tables with data on the sexo-relational profile of participants and the results of a MANOVA, replicating the results of Frías et al. (2017), who also presented their findings in a table as well as the bivariate associations of the variables in the regression models.

Moreover, we visualized the interactions of the moderation analyses in the figures 1-3.

**Supplementary table 1.**

Descriptive data on the sexo-relational profile of the participants.

|  | BPD | | | HC | | |
| --- | --- | --- | --- | --- | --- | --- |
|  | *N* | *M* | *SD* | *N* | *M* | *SD* |
| Age at initial sexual contact | 98 | 15.67 | 3.11 | 115 | 15.98 | 4.73 |
| Number of sexual partners lifetime | 114 | 9.91 | 4.68 | 115 | 8.24 | 4.08 |
|  | *N* | *n* | *%* | *N* | *n* | *%* |
| Sex, female | 50 | 50 | 100 | 55 | 55 | 100 |
| Gender identity, female | 50 | 43 | 86.0 | 55 | 55 | 100 |

**Supplementary table 2.**

Childhood trauma in women with BPD with masochistic fantasies with and without suffering from these.

|  | BPD with  masochistic fantasies (*n*=84) | | Difference | | Effect size |
| --- | --- | --- | --- | --- | --- |
| Variables | Suffering (*n*=47) | Not suffering (*n*=42) | Statistic | *p* | *η²* |
| *CTQ* |  |  |  |  |  |
| Emotional abuse | 3.34 (1.05) | 3.44 (0.87) ^a^ | *F*=0.37 | .547 | .004 |
| Physical abuse | 2.17 (1.17) | 1.81 (1.15) | *F*=2.22 | .140 | .026 |
| Sexual abuse | 3.13 (1.06) ^b^ | 2.24 (1.24) ^a^ | *F*=12.40 | .001 | .131 |
| Emotional neglect | 2.94 (1.17) | 3.00 (1.15) | *F*=0.12 | .735 | .001 |
| Physical neglect | 2.63 (1.22) ^a^ | 2.33 (1.20) | *F*=1.84 | .179 | .022 |

***Note***. BPD = Borderline personality disorder, CTQ = Child trauma questionnaire. Values range between 5 and 35. a: missing patient data n = 1. b: missing patient data n = 2.

**Supplementary table 3.**

Bivariate associations of the independent variables in the binary logistic regression model predicting BDSM 12 months prevalence (see Table 3).

|  | CSA | BSL | BSL-S | SRS | age |
| --- | --- | --- | --- | --- | --- |
| CSA | 1 | .420** | .230* | .017 | .154 |
| BSL |  | 1 | .436** | -.021 | .082 |
| BSL-S |  |  | 1 | .183 | -.056 |
| SRS |  |  |  | 1 | -.151 |
| age |  |  |  |  | 1 |

***Note.*** *N*=103. ^*^*p*<.05 ^**^*p*<.001.

**Supplementary table 4.**

Bivariate associations of the independent variables in the multiple linear regression models predicting distress from MF in sexual fantasies, masochistic fantasies, and sexual behavior (see Table 6).

|  | CSA | BSL | BSL-S | SRS | SM-Introj | age |
| --- | --- | --- | --- | --- | --- | --- |
| CSA | 1 | .554** | .327* | .017 | .114 | .165 |
| BSL |  | 1 | .437** | -.079 | .125 | .099 |
| BSL-S |  |  | 1 | .158 | -.056 | -.024 |
| SRS |  |  |  | 1 | -.151 | -.119 |
| SM-Introj |  |  |  |  | 1 | .116 |
| age |  |  |  |  |  | 1 |

***Note.*** *N*=79. ^*^*p*<.05 ^**^*p*<.001.

**Supplementary table 5.**

Bivariate associations of the independent variables in the moderation analyses to predict distress from MF in sexual fantasies, masochistic fantasies, and sexual behavior in women with BPD (see Table 7).

|  | CSA | SM-Introj | age |
| --- | --- | --- | --- |
| CSA | 1 | .115 | .146 |
| SM-Introj |  | 1 | .098 |
| age |  |  | 1 |

***Note.*** *N*=83.

**Supplementary figure 1.**

Visualization of the interaction in the moderation analysis to predict distress from MF in sexual fantasies in women with BPD (see table 7).


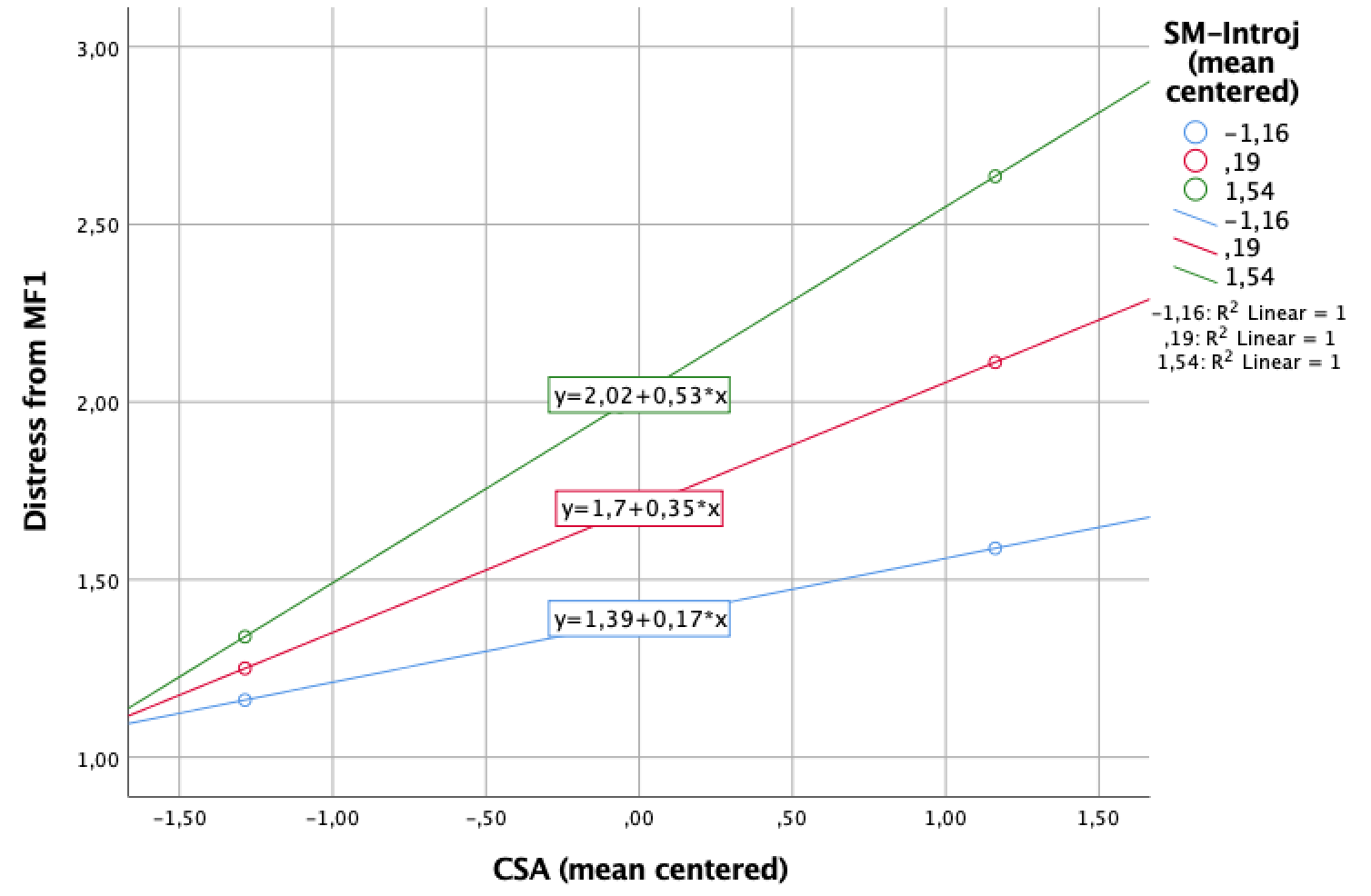


**Supplementary figure 2.**

Visualization of the interaction in the moderation analysis to predict distress from MF in masturbation fantasies in women with BPD (see table 7).


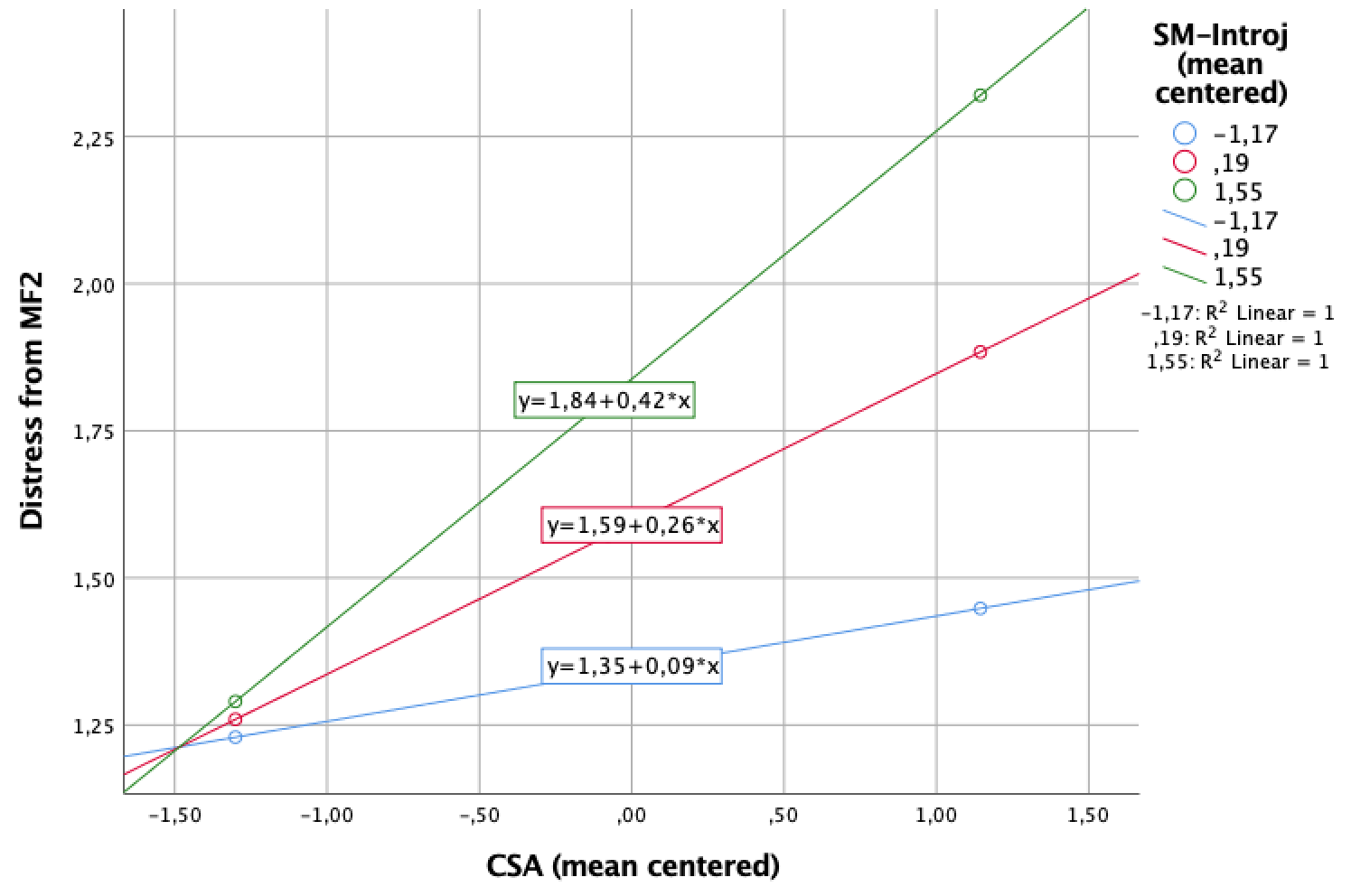


**Supplementary figure 3.**

Visualization of the interaction in the moderation analysis to predict distress from MF in sexual fantasies in women with BPD (see table 7).


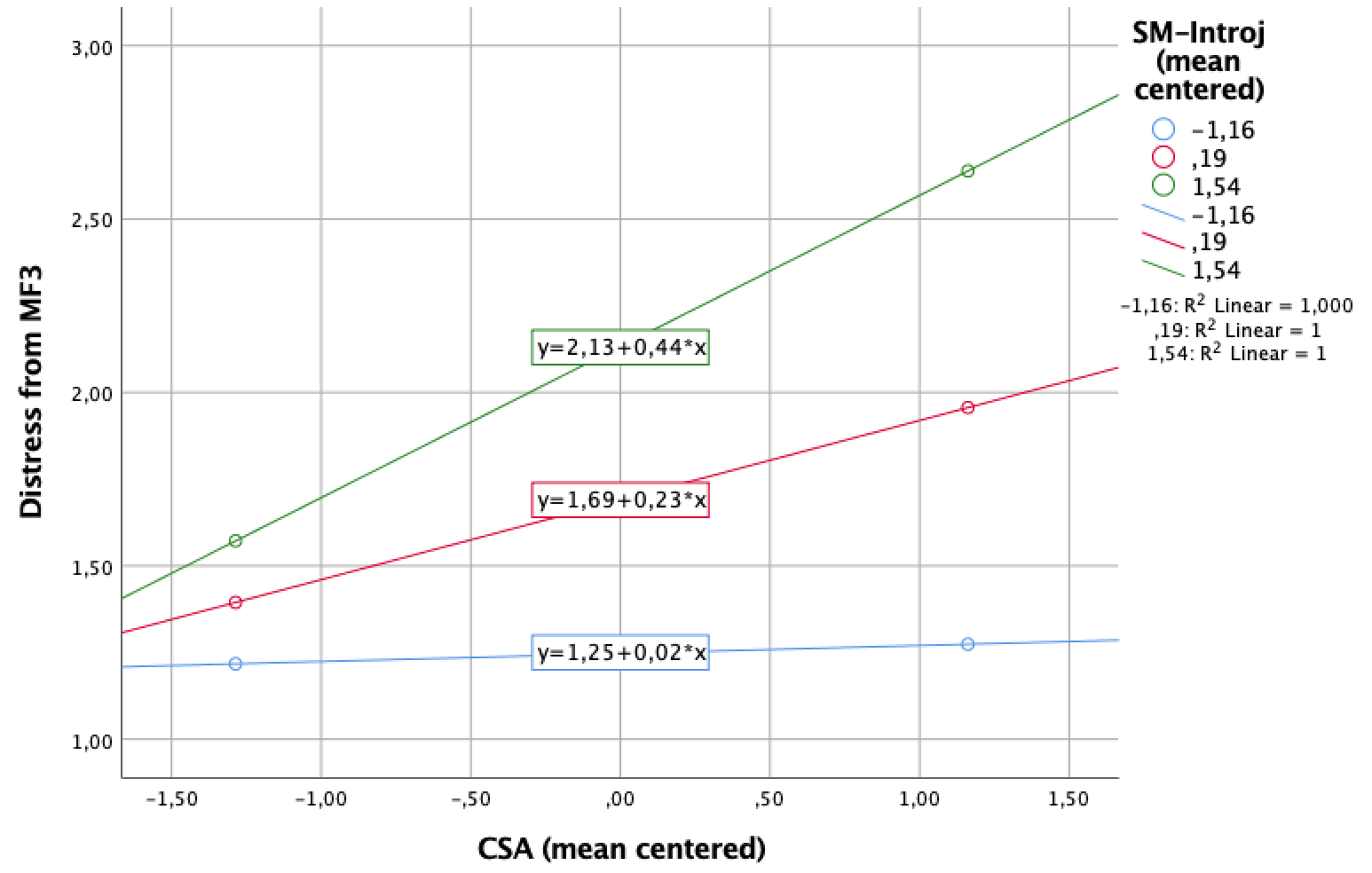

Supplement: Supplementary file 2 — Supplementary Material 2 [file 40479_2025_283_MOESM2_ESM.docx]
